# Supplementary material for: Genome of Pythium myriotylum Uncovers an Extensive Arsenal of Virulence-Related Genes among the Broad-Host-Range Necrotrophic Pythium Plant Pathogens
Source: Microbiol Spectr. 2022 Aug 10;10(4):e02268-21. doi: 10.1128/spectrum.02268-21 (PMC9430622; doi:10.1128/spectrum.02268-21)
Supplement: Supplemental file 1 — Supplemental material. Download spectrum.02268-21-s0001.pdf, PDF file, 2.1 MB [file spectrum.02268-21-s0001.pdf]

Table S1. BUSCO completeness statistics for the *P. myriotylum* SWQ7 genome assembly and for the gene model set predicted.

category	number	percentage
BUSCO analysis of genome assembly		
Complete BUSCOs (C)	224	C:95.7%
Complete and single-copy BUSCOs (S)	216	S:92.3%
Complete and duplicated BUSCOs (D)	8	D:3.4%,
Fragmented BUSCOs (F)	1	F:0.4%,
Missing BUSCOs (M)	9	M:3.9%
Total BUSCO groups searched	234	n:234
BUSCO analysis of gene model set predicted		
Complete BUSCOs (C)	223	C:95.3%
Complete and single-copy BUSCOs (S)	210	S:89.7%
Complete and duplicated BUSCOs (D)	13	D:5.6%
Fragmented BUSCOs (F)	2	F:0.9%
Missing BUSCOs (M)	9	M:3.8%
Total BUSCO groups searched	234	n:234

Table S2 (separate Excel file). **Ortholog analysis using MCL of *P. myriotylum* genes and the genes of other *Pythium* species.** Core genes are those that are present in all ten genomes, dispensable genes are those that are present in two or more genomes and unique genes are present in only one of the ten genomes.

Table S3 (separate Excel file). **Summary of the gene ontology (GO) enrichment analysis from *P. myriotylum* compared to the other *Pythium* genomes.** The enrichment or purification (underrepresentation) is in *P. myriotylum* versus (A) all *Pythium* species, (B) plant-pathogenic *Pythium* species and (C) non-plant pathogenic *Pythium* species. Only terms that were significantly enriched or purified after the Bonferroni correction were considered.

Table S4 (separate Excel file). I**nterspersed repeat analysis of the *P. myriotylum* SWQ7 genome.** (A) Summary of the repeats identified and (B) list of individual repetitive sequences in GFF file format. The analysis was run using RepeatMasker version 4.1.1 in the default mode. *Most repeats fragmented by insertions or deletions have been counted as one element.

Table S5 (separate Excel file). **Summary of the tandem clusters in *P. myriotylum*.** The genes found in these tandem clusters are listed alongside any Pfam annotation for the genes in a tandem cluster. The average percentage protein identity and average ratio of non-synonymous (dN) to synonymous (dS) substitutions amongst the members of a tandem array are also listed.

Table S6 (separate Excel file). **Summary of all CAZy families in *P. myriotylum* and other *Pythium* species.** The CAZy families were annotated using dbCAN2 [65]. Putative activities and plant substrate are listed for those CAZymes that are putative plant cell wall degrading enzymes (PCWDEs).

Table S7 (separate Excel file). **Transcriptomics dataset for *P. myriotylum* isolates infecting ginger leaves.** Summary of gene models and functional annotations for *P. myriotylum* genes and the RNAseq dataset for SWQ7 and SL2 isolates from control and during infection of ginger leaves.

Table S8 (separate Excel file). **Summary of the gene ontology (GO) enrichment analysis of the differentially expressed genes during infection of ginger leaves.** The enrichment for *P. myriotylum* SWQ7 (A and B) or *P. myriotylum* SL2 (C and D) up and down regulated genes is listed.

Table S9 (separate Excel file). **Summary of the selective pressure analysis of the *P. myriotylum* type I NLP proteins.** The analysis was performed using the yn00 program implemented in the PAML package. The omega value is the ratio of dN/dS (non-synonymous/synonymous) substitutions where a ratio < 1 indicates purifying selection, = 0 indicates neutral selection, and > 1 indicates diversifying selection.

Table S10 (separate Excel file). **Summary of primer sequences used in this study.**

A

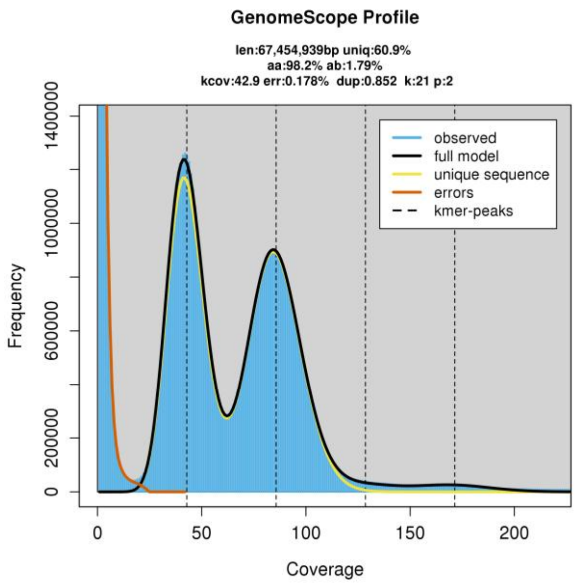

B

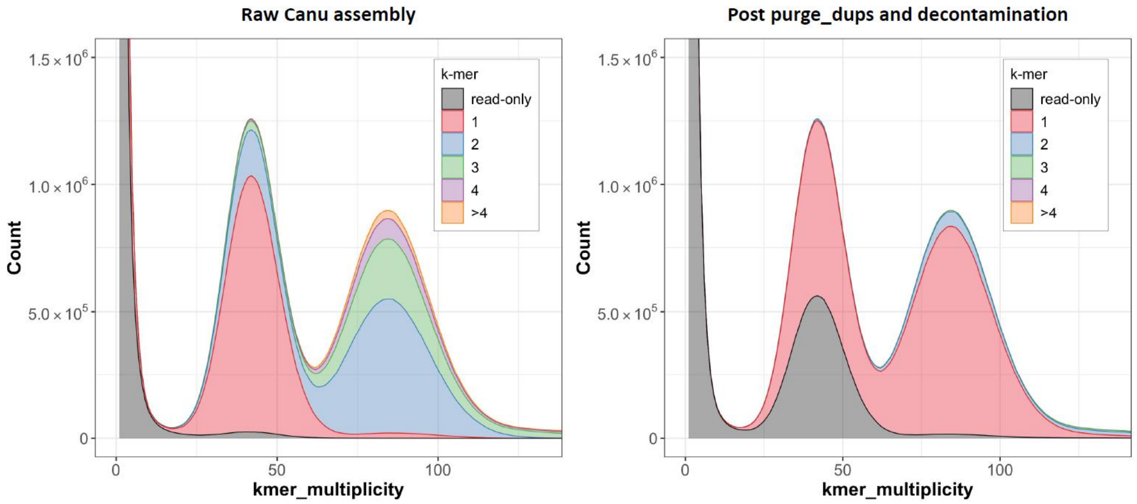

Fig. S1. **Haploid genome assembly size estimation analysis.** (A) GenomeScope analysis of k-mers from second generation sequencing of *P. myriotylum* SWQ7 genome. (B) Merqury analysis of k-mers of the raw assembly (considered the diploid assembly) and of the assembly after removal of duplicate regions and decontamination.

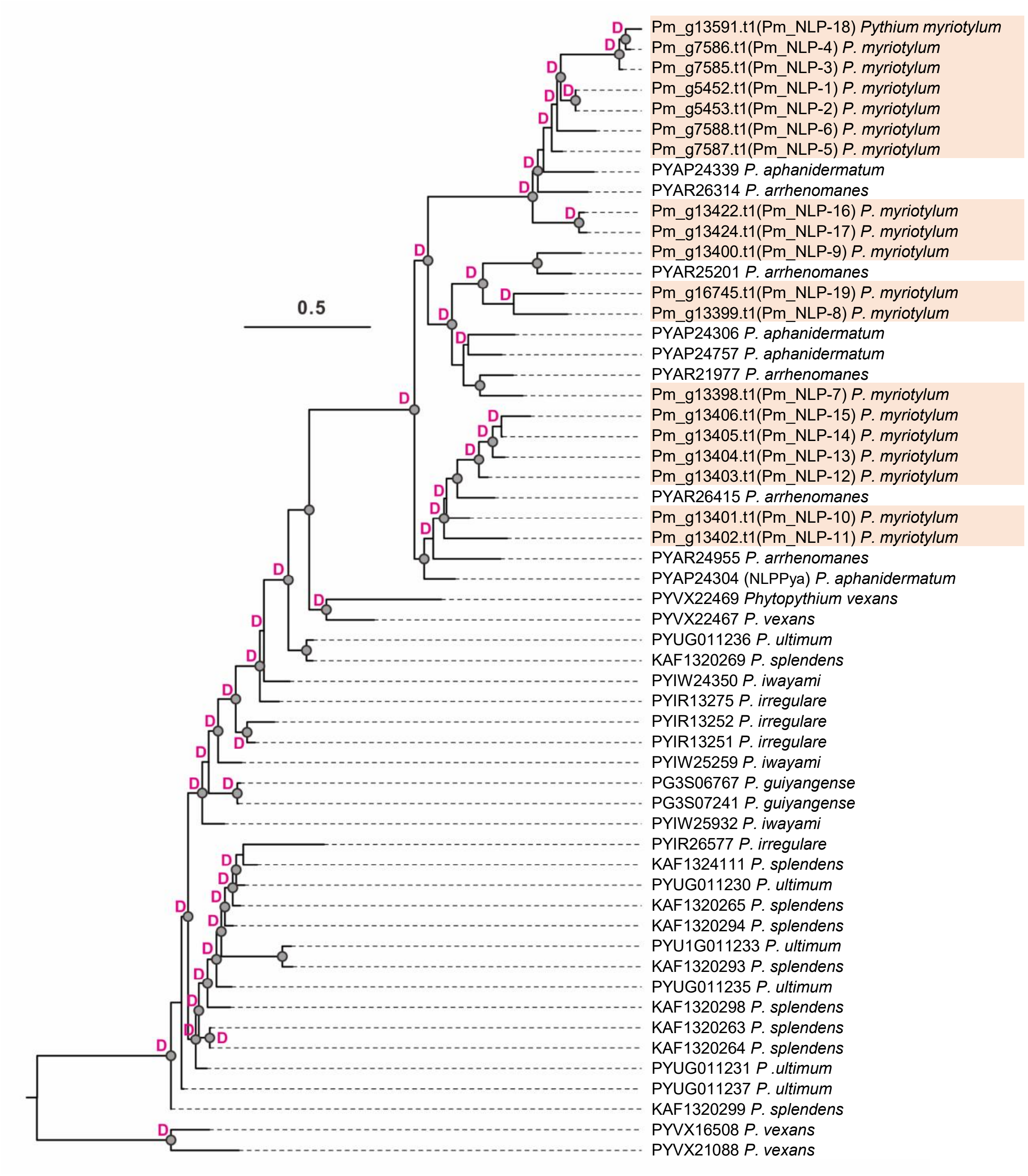

Fig. S2. **NOTUNG analysis of the evolution of type I NLP proteins in Pythium species.** The letter D denotes the putative gene duplication events revealed by the NOTUNG analysis. The P. myriotylum gene IDs are highlighted. The maximum-likelihood (ML) phylogram of NLPs was constructed using IQ-TREE (bootstrap replicate, n = 1000). Circles at the nodes indicate IQ-TREE ultrafast bootstrap values of > 70.

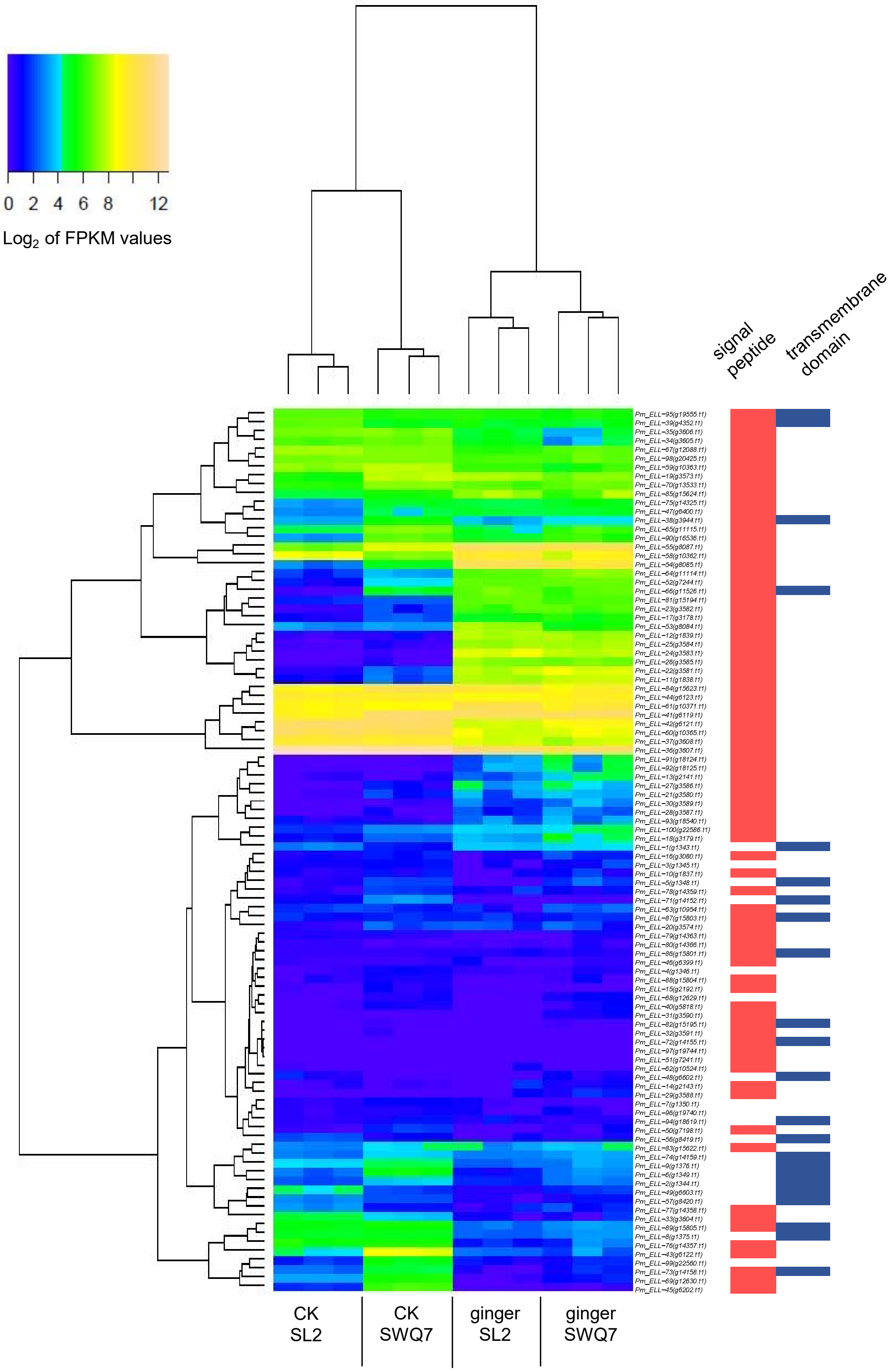

Fig. S3. **Expression levels of *P. myriotylum* elicitin-like (ELL) genes before and after infection of ginger leaves.** The hierarchical clustering was performed on the $\log_2$ FPKM values. The presence of a signal peptide and transmembrane domain is also indicated beside the gene name.

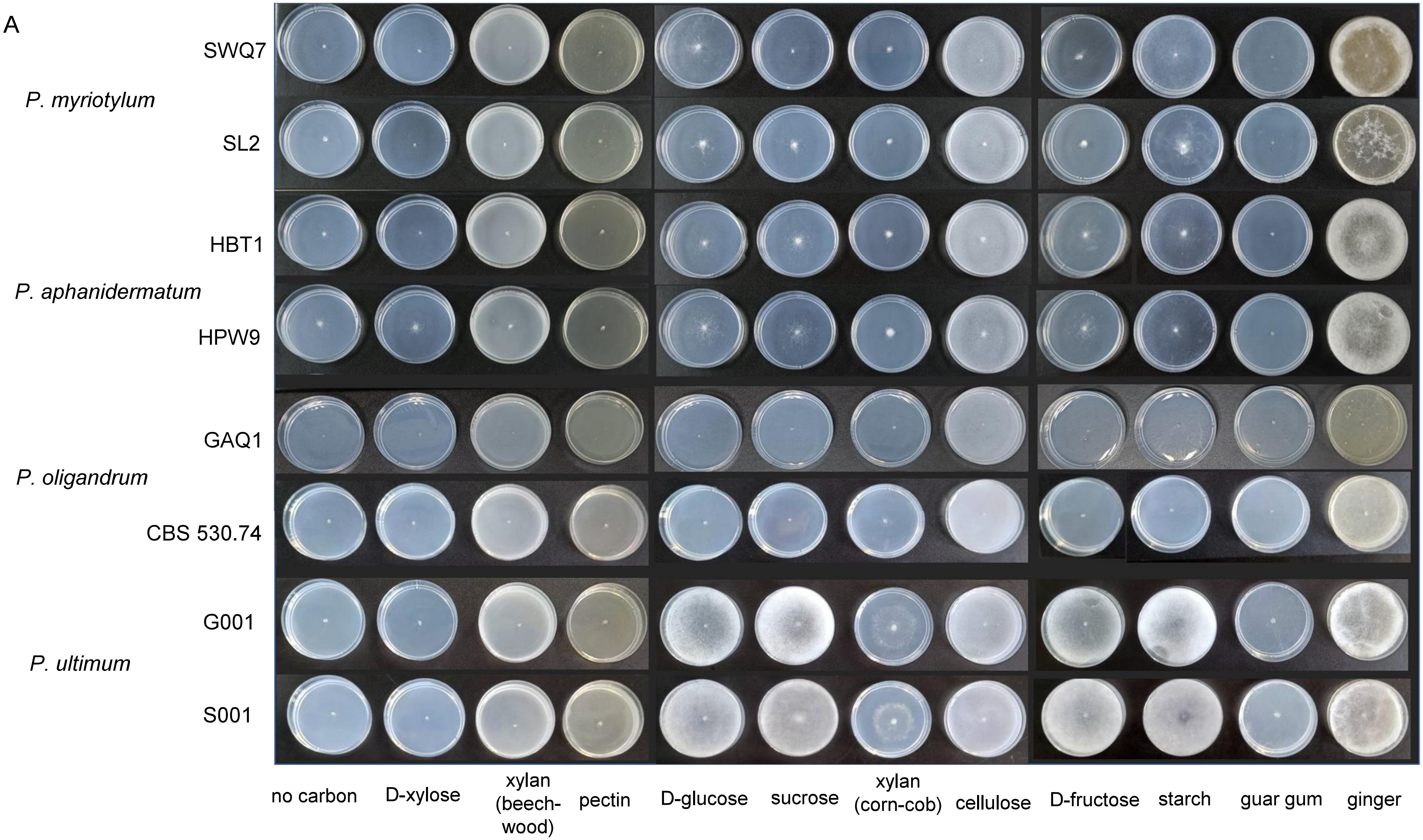

Fig. S4. Growth profiling of *Pythium* spp. on a range of plant biomass related carbon sources. (A) Images taken after 5 d showing the comparison of the growth of two *P. myriotylum* isolates to the growth of three other *Pythium* spp. on a subset of the simple and complex carbon sources. See next sheet for parts (B) and (C).

B

carbon source	*P. myriotylum*		*P. aphanidermatum*		*P. oligandrum*		*P. ultimum*	
	SWQ7	SL2	HBT1	HPW9	GAQ1	530.74	S001	G001
V8 juice	5±0	5±0	5±0	5±0	2.3±0.6	2±0	5±0	5±0
no carbon source	1±0	1±0	1±0	1±0	1±0	1±0	1.3±0.6	1.6±0.6
D-glucose	1±0	1±0	1±0	1±0	1±0	1±0	1.6±0.6	2±0
D-fructose	1±0	0±0	1±0	1±0	1±0	1±0	1±0	1±0
D-galactose	1±0	1±0	1±0	1±0	1±0	1±0	1±0	1±0
D-mannose	1±0	1±0	3±0	1±0	1±0	1±0	1±0	1±0
D-xylose	1±0	1±0	2±0	1±0	1±0	1±0	1.3±0.6	2±0
L-arabinose	1±0	1±0	1±0	2±0	1±0	1.6±0.6	2±0	2±0
L-rhamnose	1±0	1±0	3±0	1±0	1±0	1±0	2±0	2.6±0.6
cellobiose	3±0	1±0	1±0	1±0	1±0	1±0	1.6±0.6	3±0
sucrose	1±0	1±0	3±0	1±0	1±0	1±0	2±0	2±0
D-galacturonic acid	1±0	1±0	1±0	1±0	1±0	1±0	1±0	2.3±1.2
xylan (from corncob)	1±0	0±0	1±0	1±0	0.6±0.6	1±0	1±0	1±0
xylan (from beechwood)	1±0	1±0	1±0	3±0	1±0	1±0	2±0	2±0
guar gum	3±0	4±0	4±0	4±0	1±0	4±0	4.3±0.6	4.3±0.6
starch	1±0	1±0	3±0	3±0	1±0	1±0	3±0	2±0
pectin	2±0	1±0	4±0	3±0	1.6±0.6	3±0	4±0	4.6±0.6
cellulose	0±0	1±0	1±0	3±0	1±0	2±0	1.3±0.6	3±0
ginger rhizome powder	3±0	2±0	5±0	5±0	1±0	1±0	4.6±0.6	5±0

C

carbon source	*P. myriotylum*		*P. aphanidermatum*		*P. oligandrum*		*P. ultimum*	
	SWQ7	SL2	HBT1	HPW9	GAQ1	530.74	S001	G001
V8 juice	3±0.1	2.9±0.1	4.9±0.1	4.8±0.2	5.6±0.2	4.6±0.3	5.9±0.1	5.4±0
no carbon source	0.7±0	0.6±0.1	0.6±0.1	1.7±0.3	1±0.2	3.3±0.1	2.4±0.2	2±0.2
D-glucose	1±0.1	0.7±0.1	1.8±0.1	2.7±0.2	2.1±0.4	3±0.1	2.8±0.3	2.7±0.2
D-fructose	1.2±0.2	0.4±0.1	1.9±0.1	1.9±0.1	0.7±0.3	2.2±0.2	1.8±0.2	1.7±0.2
D-galactose	0.5±0	0.7±0.1	1±0.2	2.1±0.2	1.9±0.4	2.7±0.4	2.3±0.1	2.4±0.2
D-mannose	0.8±0.2	0.5±0.1	2.4±0.2	1.9±0.3	1.4±0.2	1.7±0.1	1.5±0.1	1.3±0.1
D-xylose	0.5±0	0.6±0.1	1.6±0.2	2.6±0.2	1.7±0.2	3.4±0.1	2.3±0.3	2.5±0.2
L-arabinose	0.7±0.1	0.8±0.1	1.4±0.1	2.4±0.2	2.1±0.3	3.8±0.3	2.7±0.1	2.7±0.2
L-rhamnose	0.5±0	0.6±0	2.3±0.3	2.2±0.3	2.4±0.3	3.4±0.1	3.3±0	2.9±0.1
cellobiose	0.8±0	0.6±0.1	1.7±0.1	1.6±0.1	2.3±0.4	3.1±0.4	2±0.2	2.3±0.1
sucrose	0.6±0.1	0.5±0	2.4±0.1	2.1±0.2	2.1±0.2	2.9±0.3	2.5±0.2	2.6±0.1
D-galacturonic acid	0.8±0.1	0.7±0.2	0.9±0.2	1.6±0.2	1.9±0.3	3.1±0.1	2.7±0.3	2.4±0.3
xylan (from corncob)	0±0	0±0	0.5±0.1	0.5±0.1	0.2±0.2	0.9±0.3	0.7±0.1	0.5±0.1
xylan (from beechwood)	0±0	0.4±0.1	0.9±0.2	1.5±0.3	1.9±0.1	2.4±0.2	4.2±0.1	3.5±0.2
guar gum	1.2±0.1	2±0.2	5.1±0.2	5.2±0	4.5±0.1	4.1±0.1	5.3±0.1	5.3±0
starch	0.6±0	0.5±0	3.5±0.1	3.2±0.2	3.6±0.2	3.5±0.1	4.6±0.1	4.2±0.2
pectin	0.3±0.1	0.3±0	3.7±0.1	3.7±0.1	1.9±0.6	2.2±0.1	4.5±0.1	4.4±0.3
cellulose	0±0	0±0	1±0	1.7±0.1	2.2±0.1	2.9±0.5	1.6±0.2	1.7±0.2
ginger rhizome powder	0±0	0.6±0.1	3.7±0	3.7±0.1	1.3±0	1.1±0.1	4±0.1	3.9±0.2

Fig. S4. Growth profiling of *Pythium* spp. on a range of plant biomass related carbon sources. (A) Images taken after 5 d showing the comparison of the growth of two *P. myriotylum* isolates to the growth of three other *Pythium* spp. on a subset of the simple and complex carbon sources. (B) Average scoring ± standard deviation (n = 3) of the growth after 3 d from a scale of 0 to 5 where 0 = limited growth around plug, 1 = growth does not cover entire agar surface or gaps in covering, 2 = growth covering the entire plate without gaps, 3 = also growth of aerial hyphae around plug, 4 = also growth of aerial hyphae at the edge of the plate, and 5 = dense growth considered to have overgrown the plate. (C) Colony diameter in centimetres ± standard deviation (n = 3) measured before the colony on the carbon source where growth was fastest (V8 juice) reached the edge of the Petri dish.

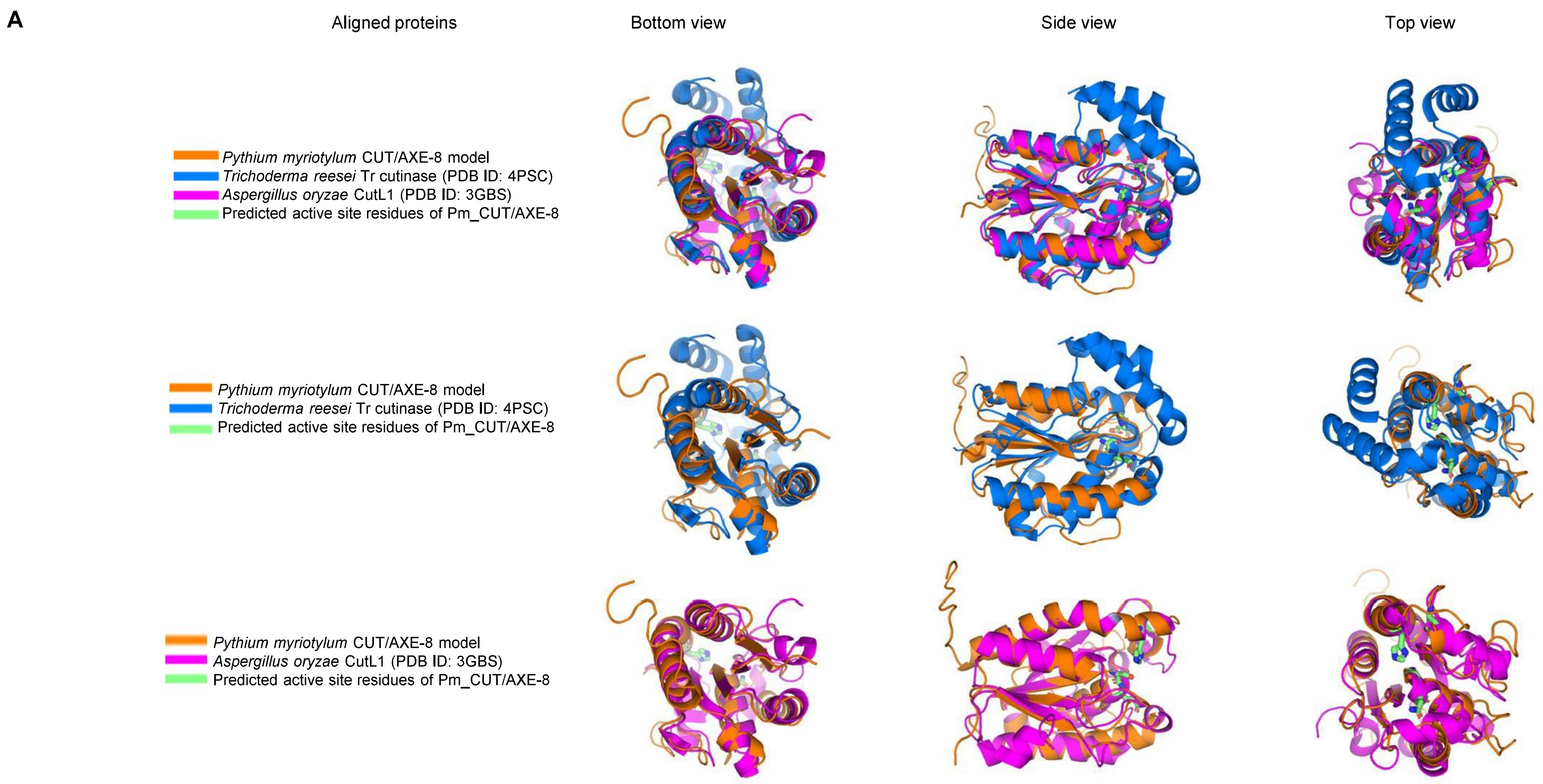

Fig. S5. Protein 3-D structure modelling of Pm_CUT/AXE-8. (A) Structural alignment of the Pm_CUT/AXE-8 model (orange cartoon) and close structural homologs *Trichoderma reesei* Tr cutinase (blue ribbon, PDB ID: 4PSC) and *Aspergillus oryzae* CutL1 (fuchsia ribbon, PDB ID: 3GBS) and visualization of secondary structural elements of the model with predicted active site residues (green sticks), (B) hydropathy plot of Pm_CUT/AXE-8 using Kyte-Doolittle hydropathy parameters including the signal sequence (first 20 amino acids), (C) the structure of Pm_CUT/AXE-8 colored based on the hydropathy scores calculated with the ConSurf server, and (D) the structure of Pm_CUT/AXE-8 colored based on the evolutionary conservation score analysis calculated with ConSurf server.

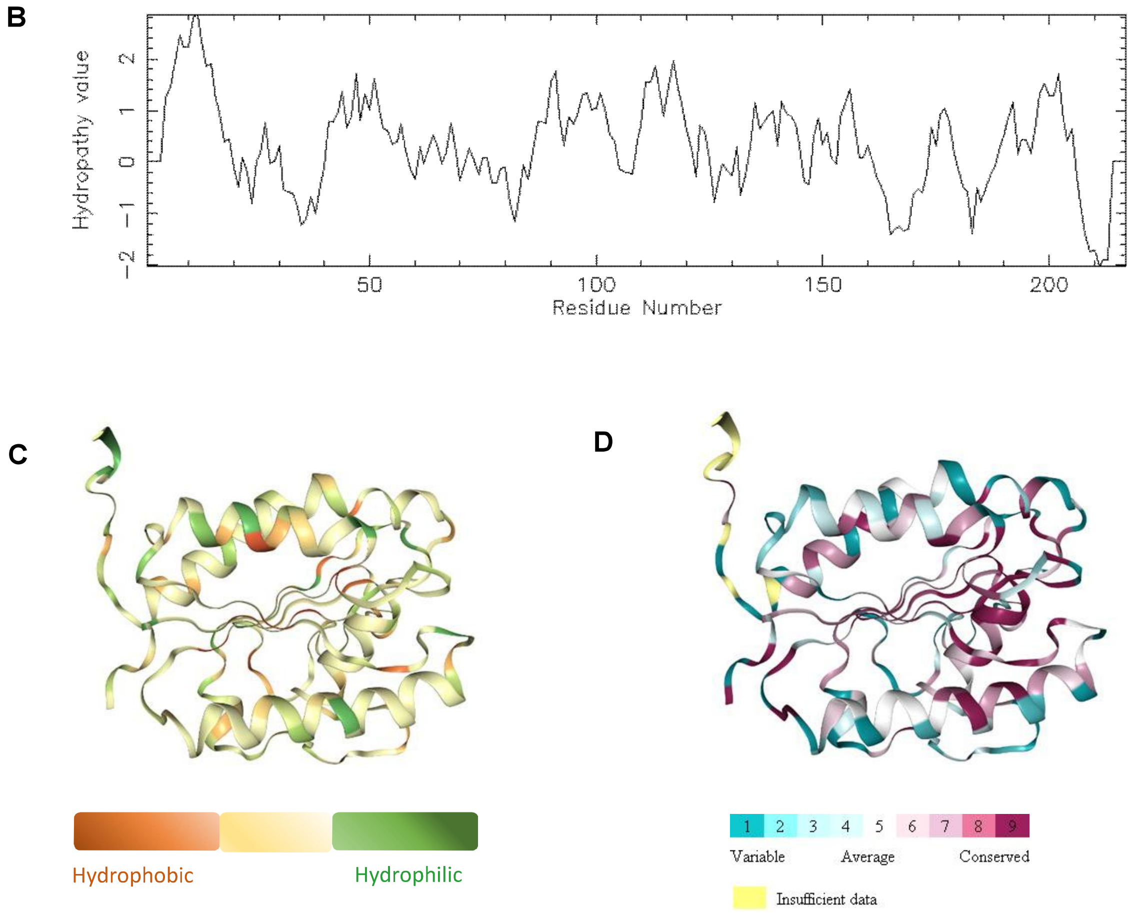

Fig. S5. Protein 3-D structure modelling of Pm_CUT/AXE-8. (A) Structural alignment of the Pm_CUT/AXE-8 model (orange cartoon) and close structural homologs *Trichoderma reesei* Tr cutinase (blue ribbon, PDB ID: 4PSC) and *Aspergillus oryzae* CutL1 (fuchsia ribbon, PDB ID: 3GBS) and visualization of secondary structural elements of the model with predicted active site residues (green sticks), (B) hydropathy plot of Pm_CUT/AXE-8 using Kyte-Doolittle hydropathy parameters including the signal sequence (first 20 amino acids), (C) the structure of Pm_CUT/AXE-8 colored based on the hydropathy scores calculated with the ConSurf server, and (D) the structure of Pm_CUT/AXE-8 colored based on the evolutionary conservation score analysis calculated with ConSurf server.

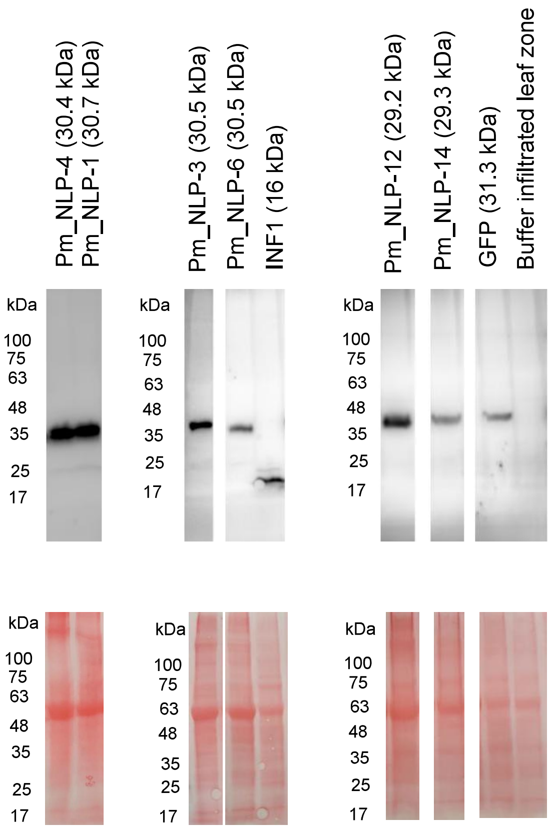

Fig. S6. Demonstration of the production of the *P. myriotylum* NLP proteins in *N. benthamiana*. The western blots were performed using anti-HA antibodies demonstrating production of Pm_NLP-1, Pm_NLP-3, Pm_NLP-4, Pm_NLP-6, Pm_NLP-12 and Pm_NLP-14 in *N. benthamiana* leaves after infiltration with *Agrobacterium* strains containing the pGR107-HA constructs. The predicted size of the HA-tagged protein (including the signal peptide for the NLPs) is indicated in parenthesis. An equal volume of the extracted protein was loaded in each lane.
